# The Anti-Toxin Treatment of Diphtheria

**Published:** 1894-12-15

**Authors:** 


### THE ANTITOXIN TREATMENT OF DIPHTHERIA.

On Friday last Dr. Sims Woodhead, at the request of the British Institute of Public Health, gave a lecture on this subject, in which he described the methods adopted in the bacteriological examination of suspected cases of diphtheria, and reviewed the evidence in favour of the antitoxin-serum treatment. The theory of the matter he reduced to its simplest terms, which in essence were as follows: The bacilli of diphtheria grow chiefly in the free surface of the membrane, practically outside the body; they do not enter the tissues; they produce in their growth toxic substances probably of the nature of enzymes, which are absorbed, and by acting on the blood produce toxins of various kinds; the general symptoms of the disease arise from the poisonous effect of these substances; for their protection, however, the tissues produce certain protective substances which neutralise these toxins and therefore may be called antitoxins; by injecting a gradually increasing dose of toxins (not the microbes but only their products) into animals these latter are made to produce a large quantity of antitoxin, some of which lodges in the serum of their blood; by injecting this highly antitoxic serum into a patient he is supplied with what is practically the lacking antidote to the poison which he is absorbing from his diphtheritic membrane, and the disease is thus rendered harmless.

Such is the theory; we must recognise, however, that finality is not yet reached; that there are lacunæ and obscure spots in our knowledge of the exact nature of both toxins and antitoxins; and that there is, therefore, even in pure investigation of the ground already won, plentiful opportunity for the useful employment of the £1,000 which, as we announced last week, has been provided for that purpose by the Goldsmiths' Company.

We cannot afford to deal with this matter as vaccination has been dealt with, and to leave it for a hundred years an isolated and empirical fact. Such knowledge as we have obtained regarding the use of antitoxins in tetanus and diphtheria will be far from its full fruition until it is made to take its place in line along with the other growing bits of knowledge we possess about all the other forms of zymotic disease, and money is well spent in hastening and helping on these life-saving investigations.

It would seem that the practical application and investigation of this treatment in England is now assured, not only by the various public bodies which have undertaken the preparation of the serum for general use, but by the action of the Metropolitan Asylums Board in accepting the offer of the Royal Colleges of Physicians and Surgeons to produce the antitoxin for use in their hospitals on certain conditions.

The protests of Lord Coleridge and the deputation which he introduced last week to the Asylums Board were of a very feeble and unconvincing character. If they meant anything they meant, on the one hand, that patients compulsorily removed to hospital should be consulted as to the details of their treatment; and, on the other, that in regard to children, who cannot enter into these puzzling questions, no treatment should be employed which had in it any element of doubt. Neither of these propositions fits in with the problems of real life.

Hospitals are managed by responsible officials with the object of curing disease. They are not hotels, in which each patient can pick his treatment from a menu according to his individual taste; and, as to experiment, all treatment, from the domestic castor oil to the scientific antitoxin, is, in a sense, experimental. We may hope, as we do, that this treatment by serum is but a step in the discovery of some general principle, and that sooner or later it will give way to something more refined; but in the meanwhile children are dying, and the evidence is strong that this stuff possesses a certain well-defined power to prevent their doing so. While, then, it is recognised as the best antidote known, while its use receives the support of physicians best qualified to judge, and while it remains the treatment to which those resort who, from their wealth, are able to get the best of everything, we think it right that it should be provided also for the poor in hospitals.

